# Genome-wide analysis of circular RNAs in prenatal and postnatal pituitary glands of sheep

**DOI:** 10.1038/s41598-017-16344-y

**Published:** 2017-11-23

**Authors:** Cunyuan Li, Xiaoyue Li, Qiman Ma, Xiangyu Zhang, Yang Cao, Yang Yao, Shuang You, Dawei Wang, Renzhe Quan, Xiaoxu Hou, Zhijin Liu, Qianqian Zhan, Li Liu, Mengdan Zhang, Shuting Yu, Wei Ni, Shengwei Hu

**Affiliations:** College of Life Sciences, Shihezi University, Shihezi, Xinjiang 832003 China

## Abstract

Circular RNAs (circRNAs) are a class of animal non-coding RNAs and play an impor-tant role in animal growth and development. However, the expression and function of circRNAs in the pituitary gland of sheep are unclear. Transcriptome profiling of circRNAs in the pituitary gland of sheep may enable us to understand their biological functions. In the present study, we identified 10,226 circRNAs from RNA-seq data in the pituitary gland of prenatal and postnatal sheep. Reverse transcription PCR and DNA sequencing analysis confirmed the presence of several circRNAs. Real-time RT-PCR analysis showed that sheep circRNAs are resistant to RNase R digestion and are expressed in prenatal and postnatal pituitary glands. GO and KEGG enrichment analysis showed that host genes of differentially expressed circRNAs are involved in the regulation of hormone secretion as well as in several pathways related to these processes. We determined that numerous circRNAs interact with pituitary-specific miRNAs that are involved in the biologic functions of the pituitary gland. Moreover, several circRNAs contain at least one IRES element and open reading frame, indicating their potential to encode proteins. Our study provides comprehensive expression profiles of circRNAs in the pituitary gland, thereby offering a valuable resource for circRNA biology in sheep.

## Introduction

Circular RNAs (circRNAs) are novel members of the non-coding RNA family in animals^1,2^. CircRNAs can be predominantly generated by back-splicing reactions, in which the 5′ and 3′ ends of linear RNAs are directly ligated^3^. Although circRNAs were first reported more than 35 years ago^4^, back-splicing is generally considered to be a rare event. High-throughput sequencing has recently revealed that circRNAs are abundantly and stably expressed in plants and animals in a tissue-specific manner. CircRNAs generally consist of exons (exonic circRNAs), but these can also arise from introns (intronic circRNAs or ciRNAs)^5,6^. Recent studies have shown that circRNAs may serve as microRNA (miRNA) sponges, sequestering miRNAs by competing with targeted mRNAs^7,8^. CDR1as is a circRNA that is derived from an antisense transcript of the CDR1 protein-coding gene. With at least 60 conserved sites for miR-7, CDR1as is thought to act as a sponge that titrates miR-7, keeping it from its other targets. In addition, some circRNAs have also been reported to function as positive regulators of RNA Pol II transcription in the cell nucleus^6^.

The mammalian pituitary gland is composed of glandular anterior and intermediate lobes. The anterior lobes produce specific hormones that are necessary for growth, fertility, lactation, pubertal development, and responses to physiological stress^9^. To relay signals from the hypothalamus, unique hormones, including prolactin (PRL), growth hormone (GH), thyroid stimulating hormone (TSH), adrenocorticotropic hormone (ACTH), and gonadotropins such as luteinizing (LH) and follicle stimulating (FSH) hormones, which are secreted by hormone-producing cell types, regulate developmental and physiological processes of the body^9,10^. Hormone synthesis and secretion within the pituitary are determined by various genes and pathways. Brinkmeier *et al*. reported that a total of 157 genes related to some key pathways are expressed in the developing anterior pituitary gland of mice^11^. Recently, Ye *et al*. identified 222 miRNAs in the anterior pituitary gland of pigs, with some possibly influencing animal postnatal growth^12^. Therefore, circRNAs may serve as novel regulators of endocrine synthesis and secretion within the pituitary gland at the transcriptional and post-transcriptional levels.

Sheep is an important animal in agriculture because of its utility in wool, meat, and milk production. The pituitary gland, as the most important endocrine organ, regulates developmental and physiological processes in sheep, including growth, reproduction, and lactation. Recent studies have shown that circRNAs are upregulated in the tissues of various animals (e.g., human, mouse, and pig) and play important roles in their growth and development^13–16^. However, expression and function of circRNAs in the pituitary gland of sheep have not been investigated in detail. CircRNA profiling of the sheep pituitary may enable us to understand the physiological functions of circRNAs in the pituitary gland. In the present study, we investigated the expression profiles of circRNAs in the prenatal and postnatal pituitary glands of sheep using Illumina HiSeq 2500 technology. The findings of this study may improve our understanding of the roles of circRNAs in the growth and development of the pituitary gland.

## Results

### Deep sequencing of circRNAs in the sheep pituitary gland

**Figure 1:**
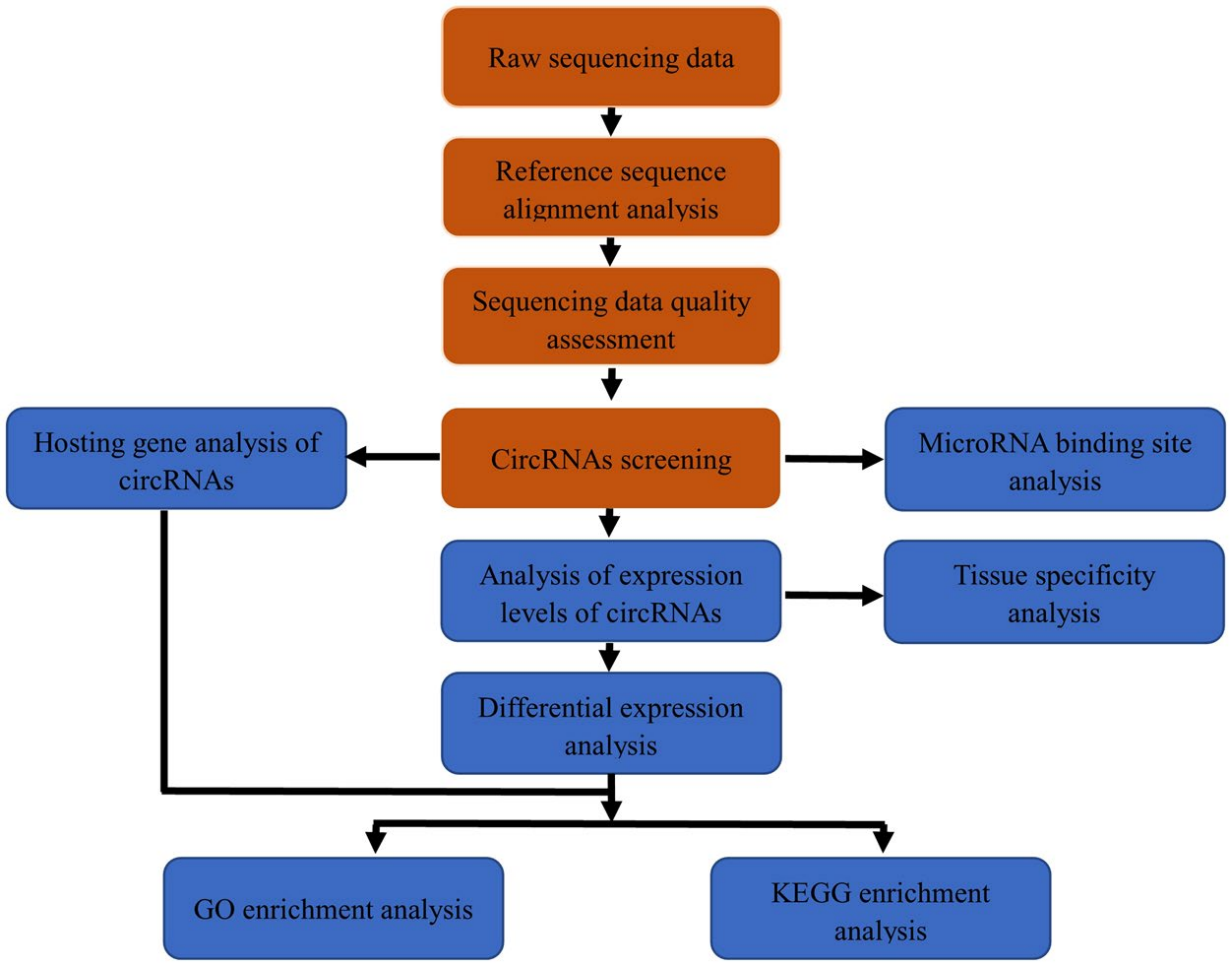
Identification pipeline for circRNAs.

**Figure 2:**
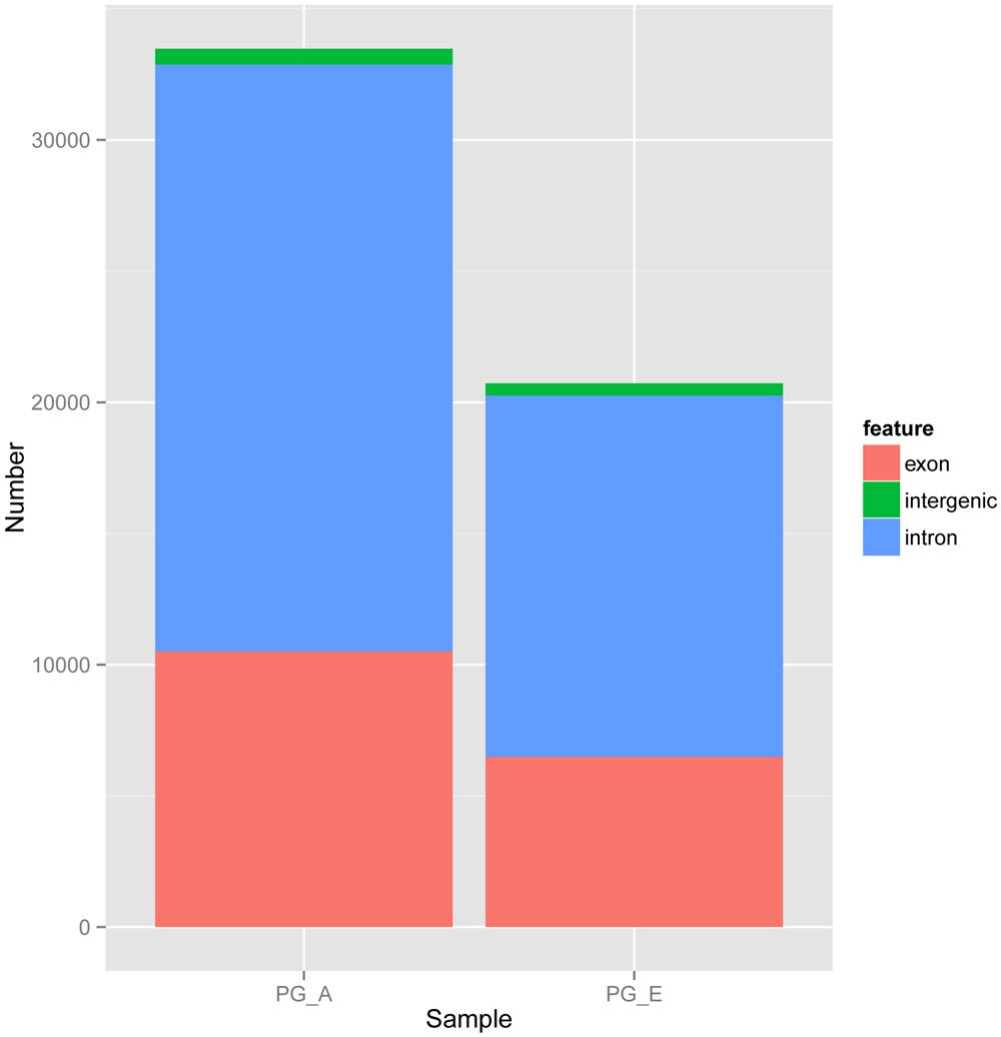
The information on circRNAs in the sheep pituitary gland generated by deep sequencing. The structure of circRNAs in the sheep pituitary gland. Green represents intergenic regions, light red indicates exons, and light blue shows introns.

To determine the identity and abundance of circRNAs in the sheep pituitary gland, paired-end ribo-minus RNA sequencing (RNA-seq) was performed as described in the pipeline (Fig. 1). The FindCirc computational pipeline was applied to detect circRNAs from two libraries^17^. We identified a total of 10,226 circRNAs from RNA-seq data of the embryo pituitary gland (PG_E) and adult pituitary gland (PG_A). Based on the genomic location of sheep, we found that these circRNAs included intronic and/or exonic sequences, whereas a small part consisted of intergenic sequences (Fig. 2). These circRNAs were distributed across 26 autosomes and the X chromosome. A full list of the circRNAs is presented in Table S2, including their annotation, chromosomal location, strand orientation, and host mRNA.

### Validation of sheep circRNAs

**Figure 3:**
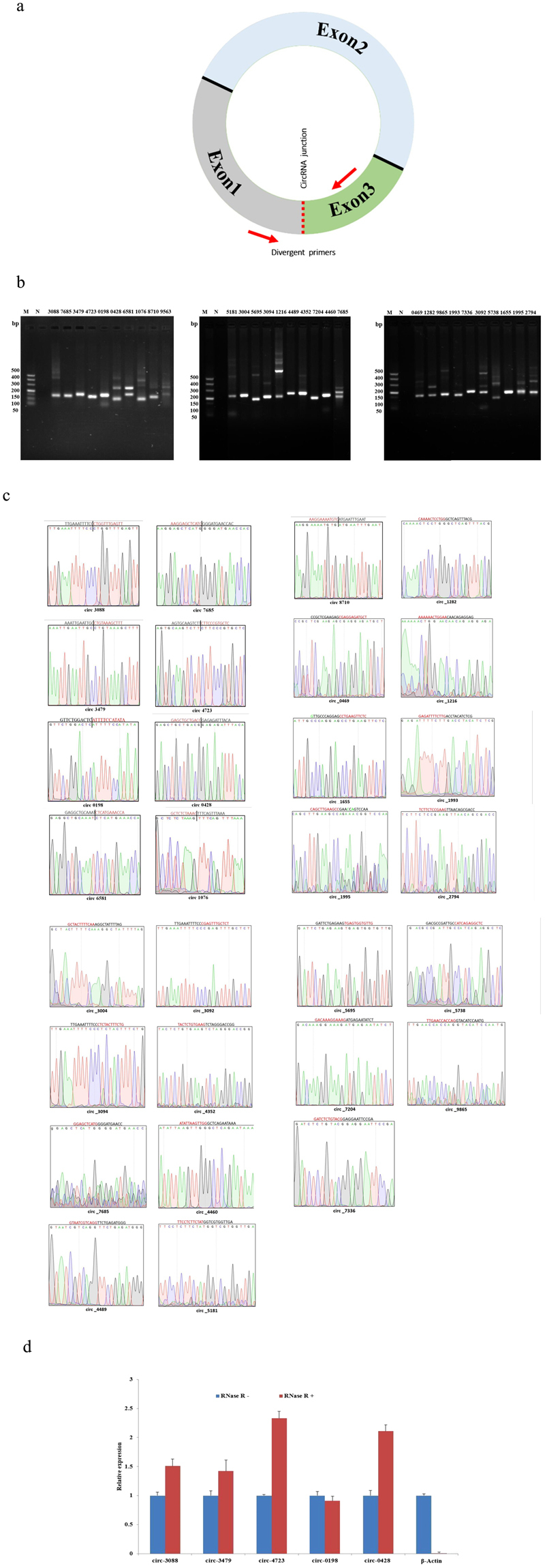
Verification of circRNAs data from RNA-seq. (**a**) Divergent primers used in the amplification of circular junctions. Red arrows represent divergent primers. (**b**) RT-PCR amplification of circRNAs with divergent primers. M is the marker (Takara DL500: 500 bp, 400 bp, 300 bp, 200 bp, 150 bp, 100 bp, and 50 bp), and N is the negative control. (**c**) Head-to-tail junctions were confirmed by DNA sequencing. (**d**) Resistance testing of circRNAs to RNase R digestion by real-time RT-PCR. β-Actin was used as a linear control. Error bars indicate ± SD.

To confirm the data on circRNAs generated from RNA-seq analysis, we further experimentally detected the expression of sheep circRNAs. To validate our sequencing data, divergent primers were designed to amplify the circular junctions (Fig. 3A). Out of 30 randomly selected circRNAs, 29 junctions (except for circ-9563) showed the expected band sizes by RT-PCR analysis (Fig. 3B). Some reactions generated nonspecific products, which could be isoforms of alternative splicing circularization^18,19^. Head-to-tail junctions were confirmed by DNA sequencing (Fig. 3C). We also tested the resistance of circRNAs to RNase R digestion by real-time RT-PCR. All five tested circRNAs showed resistance to RNase R digestion, whereas no linear β-actin mRNA was detected (sensitive to RNase R) (Fig. 3D).

### Analysis and validation of differentially expressed circRNAs

**Figure 4:**
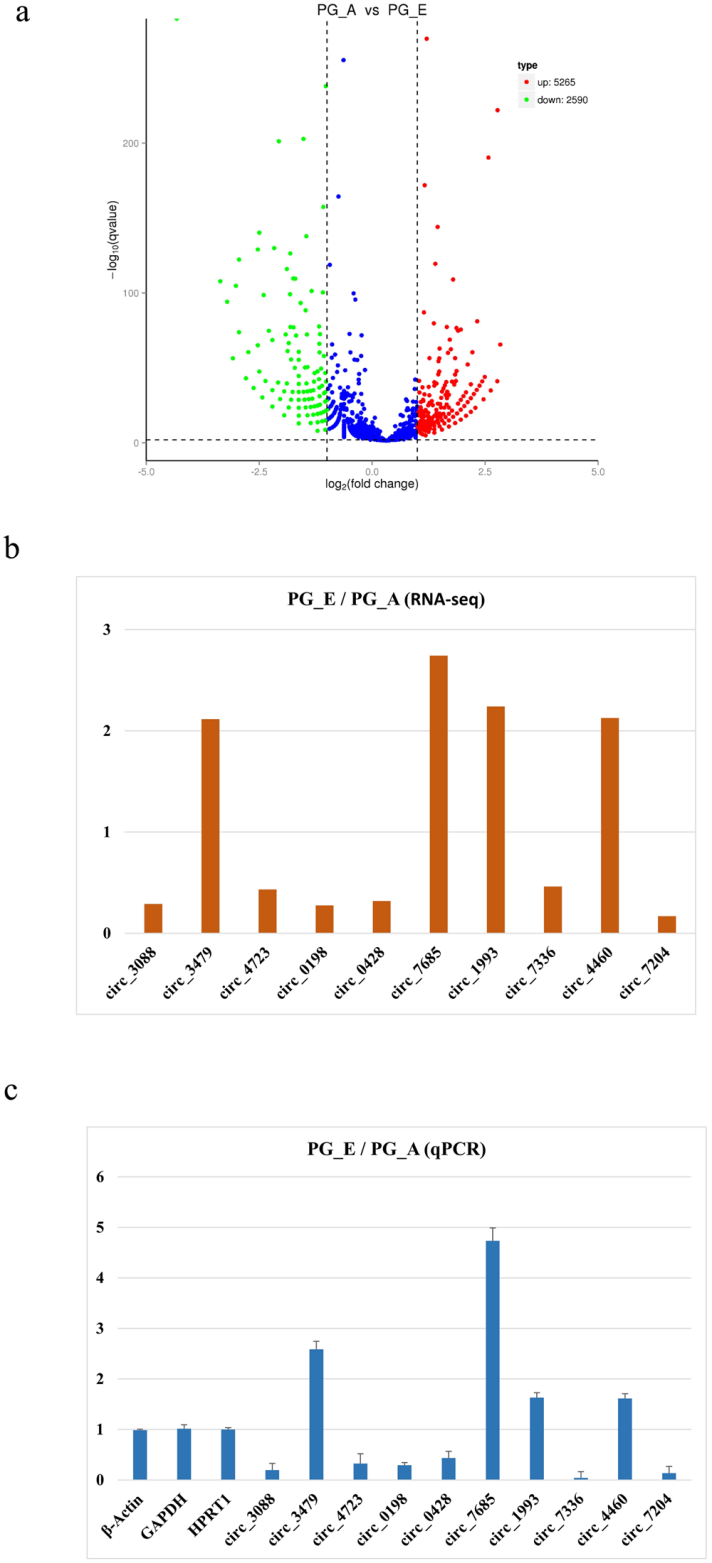
Analysis and validation of differentially expressed circRNAs in the PG_E and PG_A groups. (**a**) Volcano plot analysis of all circRNAs between PG_E and PG_A group. The logarithm of the significant difference between the two samples was analyzed by log2 (fold change) as the abscissa, and the negative logarithm-log 10 (P-value) of the P value was calculated as the ordinate (P < 0.05). Red dots indicate upregulated genes; green dots represent downregulated genes. (**b**,**c**) Change in circRNA levels between the PG_E and PG_A groups. PG_E/PG_A ratios for 10 different circRNAs based on the Illumina next-generation sequencing (RNA-seq) data (one sample) (**b**). Expression of differentially expressed genes as determined by real-time RT-PCR (three biological replicates, each done in triplicate) (**c**). Error bars indicate ± SD.

Further analysis identified 7,855 circRNAs that were differentially expressed between the embryo pituitary gland (PG_E) and adult pituitary gland (PG_A), including 5,265 upregulated and 2,590 downregulated circRNAs (Fig. 4A). We randomly selected 10 differentially expressed circRNAs and examined their expression in the PG_E and PG_A groups by real-time RT-PCR (Fig. 4B,C). Similar to our RNA-seq data, expression levels of circ-0003088, circ-0004723, circ-0000198, circ-0000428, circ-0007336, and circ-0007204 in the PG_A were higher than that in the PG_E, whereas the expression of circ-0003479, circ-0007685, circ-0001993, and circ-0004460 decreased in the PG_A. The results showed that there was a strong correlation between the real-time RT-PCR and RNA-seq data (Fig. 4B,C) and indicated that the identified circRNAs were truly differentially expressed *in vivo*.

### Enrichment of differentially expressed circRNAs

**Figure 5:**
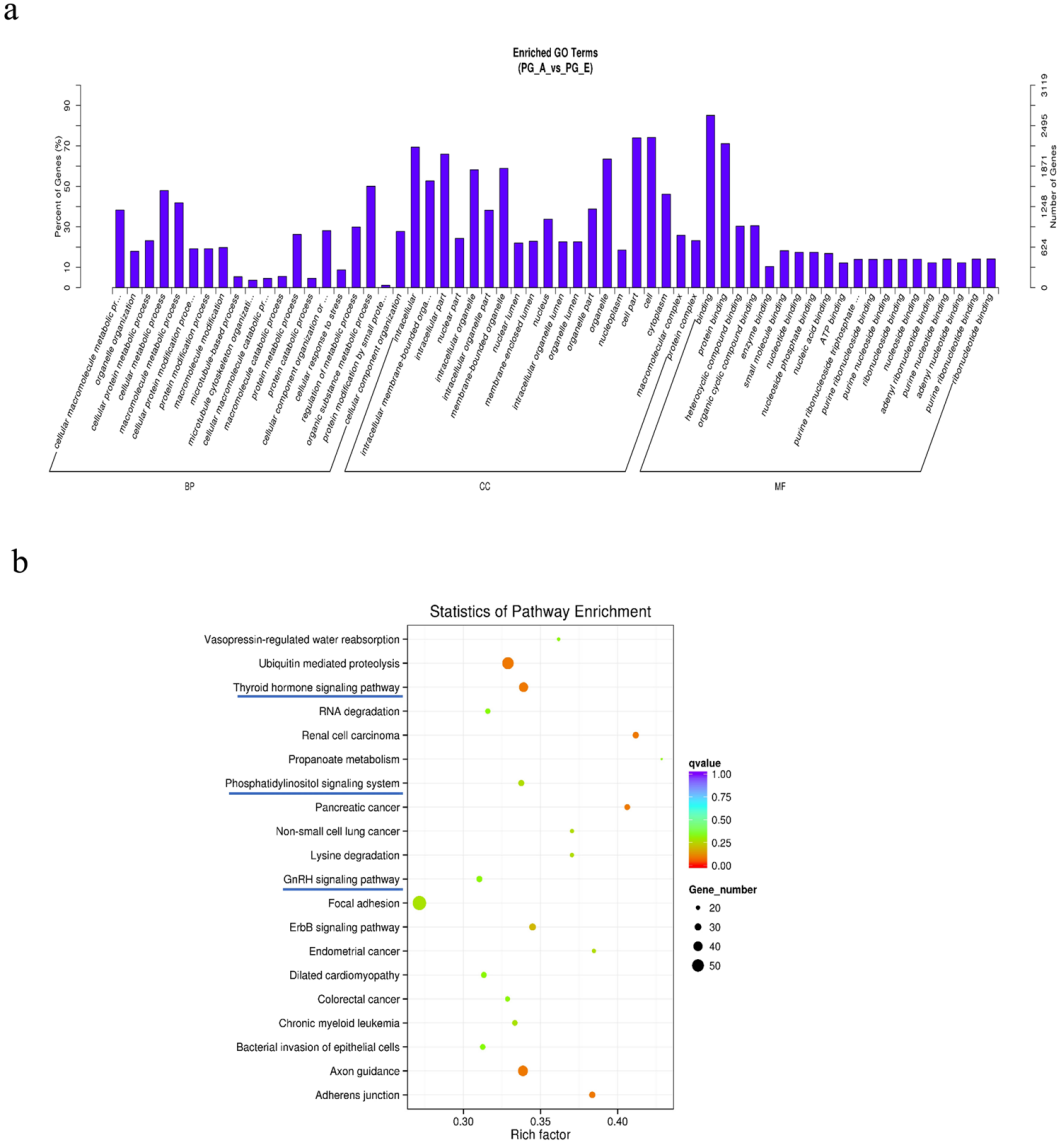
Annotations and enrichment of differentially expressed circRNAs. (**a**) GO analysis show 792 significantly (PG_A *vs*. PG_E) enriched terms (P < 0.05) in the categories of biological process, molecular function, and cellular components. (**b**) KEGG pathway analysis identified total of 270 terms that were enriched with differentially expressed circRNAs in the PG_E and PG_A groups. The thyroid hormone signaling pathway, GnRH signaling pathway, and phosphatidylinositol signaling pathway are involved in hormone secretion regulation in the adult pituitary and indicated by blue lines.

We performed enrichment analysis of host genes (Table S3) of differentially expressed circRNAs using the GO and KEGG pathways. GO analysis showed 792 significantly enriched terms (P < 0.05) in the categories of biological process, molecular function, and cellular components. Figure 5 shows the top 20 genes from GO categories that were related to metabolic process, position in cell or organelle, and function in nucleic binding, which suggest that some circRNAs are involved in basic biological regulation of the pituitary gland. KEGG pathway analysis demonstrated that 270 terms were enriched (Table S4), in which the thyroid hormone signaling pathway, GnRH signaling pathway, and phosphatidylinositol signaling pathway were involved in the regulation of hormone secretion in the adult pituitary. These results suggest that circRNAs play an important role in pituitary endocrine functions.

### Predicted functions of sheep circRNAs

Previous studies have investigated the interactions between circRNAs and miRNAs^20–24^. In the present study, miRanda and psRobot were employed to analyze the interaction between circRNAs and miRNAs. A total of 547,584 interactions between 10,226 circRNAs and various miRNAs were identified (Table S5). Recently, Zheng *et al*.^23^ reported that circRNA (circHIPK3) regulates cell growth by sponging nine miRNAs with 18 potential binding sites. Interestingly, we found that oar_circ_0000059 contained 58 potential binding sites for 9 pituitary-related miRNAs, including miR-103 (9 binding sites), miR-148 (8 binding sites), miR-150 (6 binding sites), miR-16b (6 binding sites), miR-181a (4 binding sites), miR-19b (5 binding sites), miR-23 (9 binding sites), miR-30 (7 binding sites), and miR-329 (4 binding sites). oar_circ_0000059 may be involved in development and endocrine functions of the pituitary by sponging multiple miRNAs, although these require further investigations. In addition, recent studies have shown that some circRNAs are translated into proteins by internal ribosome entry sites (IRESs)^25^. We predicted the protein-coding potential of sheep circRNAs by blasting IRES Prediction System and circRNADb: A comprehensive database for human circular RNAs with protein-coding annotations^26^. A total of 40 sheep circRNAs contained at least one IRES element and an open reading frame (OFR). These sheep circRNAs with protein-coding potential are listed in Table S6.

## Discussion

Genome assembly and annotation of some sheep breeds have been conducted in the past few years; however, transcriptome profiling of various sheep tissues has been limited. Although thousands of unique circRNAs have been identified in a wide range of cell types^27–29^, with some apparently playing important roles in growth and development, information on circRNAs in sheep remains unclear. Here, we determined that thousands of sheep genes could express circRNAs in the pituitary gland. We identified 10,226 circRNAs by high-throughput sequencing and bioinformatics analysis. We further found that there are more abundant circRNAs expressed in the sheep pituitary gland than that in the skeletal muscles and adipogenesis (Li C, oncotarget, In Prees). Interesting, Rybak-Wolf *et al*. compared circRNAs expression in human frontal cortex, thyroid gland, liverand muscle, and found an overall enrichment for circRNA expression in the nervous system. CircRNAs in the sheep pituitary gland were upregulated compared to those in the skeletal muscles and during adipogenesis (last name of author *et al*., personal communication). Similarly, Rybak-Wolf *et al*. reported that circRNAs are upregulated in the human brain compared to the thyroid, liver, and muscle^30^.

CircRNAs are a group of non-coding RNAs that act as regulators of gene expression and are involved in various developmental and physiological processes. We identified 7,855 circRNAs that were differentially expressed between the embryo and adult pituitary gland. These circRNAs may have specific biological roles in the endocrine regulation of the adult pituitary gland. The regulation of hormone secretion in the adult pituitary includes a series of changes in the expression of various genes and non-coding RNAs^11,31,32^. Gao *et al*. reported that lncRNAs in the pituitary play an important role in regulating puberty in goats^33^. Approximately 2,012 lncRNAs are differentially expressed in the pituitary gland of pubertal and prepubertal goats. Ye *et al*. observed significant changes in the miRNA and mRNA expression profiles of bama mini pigs and landrace pigs, suggesting that non-coding RNAs in the pituitary gland are involved in postnatal growth^12^. Therefore, we predict that circRNAs act as novel regulators of embryo pituitary development and endocrine regulation.

Some reports have shown that circRNAs, such as ceRNAs, influence post-transcriptional regulation by targeting miRNAs^34–37^. We have identified miRNA target sites in sheep circRNAs using miRanda and psRobot. Furthermore, we determined that various circRNAs interact with different pituitary gland-related miRNAs, such as miR-181, miR-133, miR-26, and miR-329 (Table S4). In addition, we observed that single circRNAs harbor various target sites for different miRNAs. For example, oar_circ_0000059 contained 58 potential binding sites for 9 pituitary-related miRNAs, including miR-103, miR-148, miR-150, miR-16b, miR-181a, miR-19b, miR-23, miR-30, and miR-329. However, oar_circ_0000043 included target sites for miRNA-10b, miRNA-134, miRNA-148, miRNA-181, miRNA-200, miRNA-26, and miRNA-30. Interestingly, one recent study indicated that circRNAs (circHIPK3) regulate cell growth by sponging 9 miRNAs with 18 potential binding sites^38^. Oar_circ_0000043 and oar_circ_0000059, as potential ceRNAs, may be the central regulators of pituitary gland function. This will be investigated in a future study.

Some pathways are known to be involved in the development and endocrine functions of the pituitary gland, including the BMP, FGF, WNT, SHH, and NOTCH pathways^39–43^. However, reports on the role of pituitary circRNAs are limited. In our study, the enriched KEGG and GO pathways clearly suggest that circRNAs play an important role in the basic biological regulation of the pituitary gland and hormone secretion. In particular, the thyroid hormone signaling pathway, GnRH signaling pathway, and phosphatidylinositol signaling pathway were significantly enriched, suggesting that circRNAs may be related to the regulation of hormone secretion in the adult pituitary^44^. However, the functions of circRNAs and their predicted targets should be carefully evaluated by further investigations.

In conclusion, we identified a number of circRNAs in the sheep pituitary gland and screened differentially expressed circRNAs in the prenatal and postnatal pituitary glands of sheep. Meanwhile, we showed by KEGG pathway analysis that several circRNAs are involved in endocrine regulation. Our study provides a valuable resource for understanding circRNA biology and contributes to understanding circRNA function in the pituitary gland.

## Materials and Methods

### Ethics Statement

All procedures involving animals were approved by the Animal Care Committee of Shihezi University. The study was performed in accordance with the ethical standards established in the 1964 Declaration of Helsinki and subsequent amendments.

### Collection of tissue samples

Three embryo pituitary glands from fetuses were obtained by surgery from estrus of three pregnant ewes 130 days after mating, designated as the embryo pituitary gland (PG_E) group. Three adult pituitary glands were obtained from kazakh sheep (female) that were sacrificed at a commercial slaughterhouse, designated as the adult pituitary gland (PG_A) groups. Pituitary glands were carefully dissected from each brain and immediately frozen in liquid nitrogen until RNA isolation. Total RNAs were isolated using TRIzol (Invitrogen, CA, USA) according to the manufacturer’s protocol. The quantity and purity of RNAs were assessed using a Bioanalyzer 2100 and RNA6000 Nano Kit (Agilent, CA, USA).

### RNA sequencing and quality control

The equal amounts of total RNAs (4 μg) from three adult samples were pooled to a single sample for constructing PG_A library. Similarly, the equal amounts of total RNAs (4 μg) from embryo samples were pooled to a single sample for PG_E library. A total amount of 10 μg RNA was used for each library preparation (PG_A and PG_E library). First, ribosomal RNAs were depleted using an Epicentre Ribo-zero™ rRNA removal kit (Epicentre, USA) to obtain rRNA-depleted RNAs. The rRNA-depleted RNAs were digested with Rnase R (RNR-07250, Epicentre) to remove linear RNAs, and then were subjected to TRIzol extraction. The sequencing libraries were subsequently generated using NEBNext Ultra Directional RNA library prep kit for Illumina (NEB, USA) according to the manufacturer’s protocol. Briefly, fragmentations were performed with divalent cations in NEBNext first strand synthesis reaction buffer. First-strand cDNAs were synthesized with random hexamer primers and M-MuLV reverse transcriptase (RNaseH-). Second-strand cDNAs were subsequently synthesized with DNA polymerase I and RNase H. In the reaction buffer, dNTPs with dTTP were replaced by dUTPs. The remaining over hangs were converted into blunt ends via exonuclease/polymerase activities. After adenylation of 3′ ends of DNA fragments, NEBNext Adaptor was ligated for hybridization. To select cDNA fragments of preferentially 150–200 bp in length, the library fragments were purified with AMPure XP system (Beckman Coulter, Beverly, CA, USA). The size-selected, adaptor-ligated cDNAs were treated with 3 μL of USEREnzyme (NEB, USA). PCR was conducted using Phusion High-Fidelity DNA polymerase, universal PCR primers, and index Primers. Finally, the library was purified (AMPure XP system) and then qualified by the Agilent Bioanalyzer 2100 system. The clustering of the index-coded samples was performed on a cBot Cluster Generation System using HiSeq PE Cluster Kit v4 cBot (Illumina) following the manufacturer’s recommendations. The library preparations were sequenced on an Illumina Hiseq 2500 platform and 125-bp paired-end reads were generated. Raw data (raw reads in fastq format) were first processed by a custom Perl script. Clean reads were obtained after removing the reads with more than 10% unknown bases, reads containing adapters, and reads with more than 50% of low-quality bases (whose Phred scores were <5%). Moreover, the quality of the clean reads (Q20, Q30, and GC content) was assessed. The subsequent analyses were based on the high-quality clean reads.

### CircRNA identification

The reference genome and gene annotations were downloaded from the sheep reference genome (http:/genome.ucsc.edu/). The index of the reference genome was built using Bowtie v2.0.6, and paired-end clean reads were aligned to the reference genome using TopHat v2.0.9. The unmapped reads were kept and 20-mers from the 5′ and 3′ end of these reads were extracted and aligned independently to the reference sequences by Bowtie v2.0.6. Anchor sequences were extended by find_circ^17^ such that the complete read aligned and the breakpoints were flanked by GU/AG splice sites. Then the back-spliced reads with at least two reads were annotated as circRNAs.

### Analysis of differentially expressed circRNAs

Expression level of circRNAs was normalized by transcript per million (TPM) using the following criteria^45^. The differential expression between two groups was assessed using DEGseq (version 1.20.0)^46^. P-values were adjusted by the q-values^47^, and (q-value < 0.01) and (|log2 (fold change)| >1) were set as the threshold for differential expression by default.

### Target site prediction and enrichment analysis

miRanda and psRobot were used in the analysis of the interactions between circRNAs and miRNAs^48,49^. The miRNAs were those expressed in the same samples as the circRNAs. The host genes of circRNAs were identified by using a script. We searched for the 5′ end or 3′ end of each circRNAs which accurately matched with ends of the circRNA and considered the corresponding gene of this transcript fragment as the host gene of the circRNA. Host genes of circRNAs were used in listing gene names that were submitted to the DAVID software for GO analysis^50^. A KEGG enrichment analysis of host genes of circRNAs was performed with KOBAS software^51^. Scores with P < 0.05 were considered significant for enrichment analysis.

### Coding potential analysis of circRNAs

The protein-coding potential of sheep circRNAs was predicted as previously described^26^. An IRES element is required to initiate translation of an mRNA sequence without a 5′-cap structure^52^. If a circRNA has at least one IRES element, then it may be able to encode a protein. To annotate the coding potential of sheep circRNAs, a Viral IRES Prediction System (VIPS) was employed to predict the IRES element in the sequence of each sheep circRNAs^53^. In addition, the longest ORFs were predicted for each circRNA using DNAman. Any frames with a length >300 bp were considered as an ORF. Furthermore, to verify the potential coding ability of sheep circRNAs, a public database for human circRNAs with protein-coding annotations (circRNADb) was used to screen for protein-coding circRNAs. The circRNAs, which are highly similarity to human protein-coding circRNAs, were considered as sheep circRNAs with coding potential and are listed in Table S6.

### RT-PCR analysis and DNA sequencing

Total RNAs were extracted from pituitary glands of sheep using TRIzol (Invitrogen, CA, USA). From purified RNA, cDNA was synthesized using RT-PCR Kit (Takara, Dalian, China). PCR was conducted using specific primers for circ-0003088, circ-0007685, circ-0003479, circ-0004723, circ-0000198, circ-0000428, circ-0006581, circ-0001076, circ-0008710, circ-0009563, circ-0000469, circ-0001216, circ-0001282, circ-0001655, circ-0001993, circ-0001995, circ-0002794, circ-0003004, circ-0003092, circ-0003094, circ-0004352, circ-0007685, circ-0004460, circ-0004489, circ-0005181, circ-0005695, circ-0005738, circ-0007204, circ-0009865, and circ-0007336. The primer sequence for these circRNAs are listed in Table S1. The PCR products were analyzed by gel electrophoresis. The sequences of the PCR products were compared with the sheep reference genome and RNA-seq data using DNAMAN software. PCR was conducted using the following reaction system: 10 μL of the premix (Takara, Dalian, China), 2 μL of the cDNA template, 0.6 μL of the upstream and downstream primers, respectively, and 6.8 μL of RNase-free ddH_2_O water. RT-PCR was performed using the following thermocycling conditions: an initial denaturation at 95 °C for 3 min, followed by 45 cycles at 95 °C for 10 s, 58 °C for 15 s, and 72 °C for 5 s.

### Real-time RT-PCR analysis

The expression levels of 10 circRNAs (circ-0003088, circ-0003479, circ-0004723, circ-0000198, circ-0000428, circ-0007685, circ-0001993, circ-0007336, circ-0004460, and circ-0007204) were detected by real-time RT-PCR analysis, and three housekeeping genes (β-actin, GAPDH, and HPRT1; normalized to the geometric mean of three control genes) that were stably expressed in brain tissues were used as internal reference genes^54,55^. To determine the resistance of circRNAs to RNase R digestion, total RNAs were treated with RNase R (RNR-07250, Epicentre) prior to cDNA synthesis. To validate differentially expressed circRNAs, total RNAs were directly subjected to cDNA synthesis with a RT-PCR kit (Takara, Dalian, China). Real-time PCR was performed using SYBR Green (TaKaRa Biotech, Dalian) according to the manufacturer’s protocol. The levels of circRNA expression were normalized to linear β-actin, GAPDH, and HPRT1^56^. Three independent experiments were performed on triplicate samples. Real-Time RT-PCR was conducted using the following reaction system: 10 μL of SYBR Premix DimerEraser (Takara, Dalian, China), 2 μL of cDNA, 0.6 μL of the upstream and downstream primers, respectively, and 6.8 μL of RNase-free ddH_2_O water. Real-time RT-PCR was performed with the following thermocycling conditions: an initial denaturation at 95 °C for 30 s, followed by 45 cycles at 95 °C for 5 s, 58 °C for 20 s, and 72 °C for 20 s.

### Data Availability

The data have been deposited into NCBI sequence read archive (SRA) database under accession number PRJNA 414761.

## Electronic supplementary material

Supplementary Tables S1

Supplementary Tables S2-S6
