# Supplementary material for: Genome-wide analysis of circular RNAs in prenatal and postnatal pituitary glands of sheep
Source: Sci Rep. 2017 Nov 23;7:16143. doi: 10.1038/s41598-017-16344-y (PMC5700919; doi:10.1038/s41598-017-16344-y)
Supplement: Supplementary file 1 — Supplementary Tables S1 [file 41598_2017_16344_MOESM1_ESM.doc]

**Supplementary material for**

G**enome-wide analysis of circular RNAs** [**in prenatal and postnatal**](http://xueshu.baidu.com/s?wd=paperuri%3A(9e30a81034984a8be3889223ca73f061)&filter=sc_long_sign&sc_ks_para=q%3DThe evolution ofHomo sapiens denisovaandHomo sapiens neanderthalensismiRNA targeting genes in the prenatal and postnatal brain&sc_us=17431911151666729642&tn=SE_baiduxueshu_c1gjeupa&ie=utf-8) **pituitary glands of sheep**

Cunyuan Li^1^†, Xiaoyue Li^1^†, Qiman Ma^1^, Xiangyu Zhang^1^, Yang Cao^1^, Yang Yao^1^, Shuang You^1^, Dawei Wang^1^, Renzhe Quan^1^, Xiaoxu Hou^1^, Zhijin Liu^1^, Qianqian Zhan^1^, Li Liu^1^, Mengdan Zhang^1^, Shuting Yu^1^, Wei Ni^1,*^, Shengwei Hu^1,*^

^1^College of Life Sciences, Shihezi University, Shihezi, Xinjiang, 832003, China

†These authors contributed equally to this work

*Corresponding author: Email: [niweiwonderful@sina.com](mailto:niweiwonderful@sina.com); E-mail: [hushengwei@163.com](mailto:hushengwei@163.com)

**Supplementary Table Legends:**

**Table S1.** Primer sequence for circRNAs and housekeeping genes.

**Table S2.** The information of identified circRNAs. (Provided as separate MS Excel file).

**Table S3.** The hosting gene information of identified circRNAs. (Provided as separate MS Excel file).

**Table S4.** KEGG pathway analysis demonstrated 270 terms were enriched. (Provided as separate MS Excel file).

**Table S5.** A total of 547583 interaction relationships between 10226 circRNAs and various miRNAs were found. (Provided as separate MS Excel file).

**Table S6.** CircRNAs with protein-coding potential. (Provided as separate MS Excel file).

**Table S1.** Primer sequence for circRNAs and housekeeping genes.

| Gene name | Primer sequence  (forward/reverse) | Annealing Temperature(℃) | Primer length（bp） | Amplicon size  (bp) |
| --- | --- | --- | --- | --- |
| circ-0003088 | TGAAAAGGAAAACAAACAGC | 49.25 | 20 | 147 |
|  | AGCACCAGACAAAACAAGAG | 53.35 | 20 |  |
| circ-0007685 | TTCTTTTCCAGGGACAACAA | 51.30 | 20 | 148 |
|  | ATCATACTGCTCAAGGCTCA | 53.35 | 20 |  |
| circ-0003479 | ATTGGTGAAATGGAGGATGA | 51.30 | 20 | 167 |
|  | CTTTAGGGGCTGCTGTAGAT | 55.40 | 20 |  |
| circ-0004723 | GGCTGTTCACGCAGAGAGAC | 59.50 | 20 | 140 |
|  | GGGAGATTTGTGCTGATGGAT | 55.61 | 21 |  |
| circ-0000198 | GACACAGAGAACAGGAGGAGC | 59.52 | 21 | 158 |
|  | TCCATGTGTAGCGAAACGAA | 53.35 | 20 |  |
| circ-0000428 | TGGGAATTGATCTCGTTCAT | 51.30 | 20 | 114 |
|  | GCATAAGTTGCCGTTAAGGT | 53.35 | 20 |  |
| circ-0006581 | AGCAGAAAATGAGAAAGTGGAGC | 55.99 | 23 | 169 |
|  | ACATTTCCCGCAGCCATTCT | 55.40 | 20 |  |
| circ-0001076 | TGCTCTTGGAGGTGTGGGCT | 59.50 | 20 | 112 |
|  | TTCAGATGGGAGTTTTCGGC | 55.40 | 20 |  |
| circ-0008710 | CAAAACACCAAAGAACAACG | 51.30 | 20 | 132 |
|  | TCCGAATAGCATCCTGAAGT | 53.35 | 20 |  |
| circ-0009563 | CGACGTTCAGCACATCTTG | 55.16 | 19 | 172 |
|  | ACACTTACCATCCTCCCACT | 55.40 | 20 |  |
| circ-0003094 | AGGGAATAAGCATCTCGGAA | 53.35 | 20 | 159 |
|  | AACAAGCACCAGACAAAACAA | 51.71 | 21 |  |
| circ-0005738 | TTCCTGCGAGAGACCTTTTC | 55.40 | 20 | 111 |
|  | GTTGGCTGGACTCTCACTTTC | 57.57 | 21 |  |
| circ-0001282 | ACCCTGGTAGTCAGAAGCGAT | 57.57 | 21 | 159 |
|  | TATCCCATAGAGTTTCATCAGGTC | 56.15 | 24 |  |
| circ-0007204 | GGCCATTACTTTTTCCTCTTT | 51.71 | 21 | 145 |
|  | ATAATGGAGGCAGACTTAGAGC | 55.81 | 22 |  |
| circ-0001655 | GTGCCTTTGCGTCACTGAGT | 57.45 | 20 | 164 |
|  | AACACCAGCCACCACCTTCT | 57.45 | 20 |  |
| circ-0004352 | CAACATCCGCAGCGACAC | 57.18 | 18 | 191 |
|  | CCCAGATGTTCAGGCACTT | 55.16 | 19 |  |
| circ-0004460 | CATTAACTGAGTCCCAAATACAAG | 54.44 | 24 | 170 |
|  | GCAGCTAAAACATTGGTCTCTT | 53.95 | 22 |  |
| circ-0007685 | CTCACTCTGCCTTCTTTTCCA | 55.61 | 21 | 167 |
|  | GCTTCGTTATCATACTGCTCAA | 53.91 | 22 |  |
| circ-0002794 | ATGAAGGAAAGAAAGGGGT | 50.85 | 19 | 160 |
|  | CGTCACAGAACTTGCACAG | 55.16 | 19 |  |
| circ-0005695 | GGACTTGTGTAATGTCAACTTT | 52.08 | 22 | 142 |
|  | ATGAGAGATTCTACTTTCACCTAT | 52.73 | 24 |  |
| circ-0003092 | AAGGCTCACTGATGAACTGC | 55.40 | 20 | 171 |
|  | AGCAGTCATCAGCCTGTCTT | 55.40 | 20 |  |
| circ-0001995 | GATGACCTGGACGAAGGAGAA | 57.57 | 21 | 151 |
|  | GAGGACGACACCGTTGAGC | 59.48 | 19 |  |
| circ-0000469 | TACCTGCCTTCTGCCCACA | 57.32 | 19 | 156 |
|  | GTTCTCCTTGAAGATGTCCTCCT | 57.77 | 23 |  |
| circ-0001216 | TGGCTCGGATGTTGGTGG | 57.18 | 18 | 158 |
|  | GGGGAGAGTAGCTTCCAGGATA | 59.54 | 22 |  |
| circ-0001993 | CTACTTGGGCACCACGTTTG | 57.45 | 20 | 150 |
|  | CCGTACAGGCGCATGTTG | 57.18 | 18 |  |
| circ-0003004 | GTCATTGTGGCCTTAGTGATT | 53.66 | 21 | 183 |
|  | ACCTTGAAGAGCCTGTTGAT | 53.35 | 20 |  |
| circ-0004489 | TACCTCTTGGACCCTTACG | 55.16 | 19 | 190 |
|  | AGTTTCTGGTTCCCTCTGTT | 53.35 | 20 |  |
| circ-0005181 | GCGGATGCTTGCTTCTTCAC | 57.45 | 20 | 178 |
|  | GAACGGACGGAGGTGGAGA | 59.48 | 19 |  |
| circ-0007336 | TCTTGGCTATGTGGAACTGT | 53.35 | 20 | 182 |
|  | CAATATCAAATGAATCTGTAGAAAA | 49.74 | 25 |  |
| circ-0009865 | CCTTCGAGGTCTTCCCTACG | 59.50 | 20 | 166 |
|  | TGCGGACAATGACTTGGTTT | 53.35 | 20 |  |
| GAPDH | ATGCCTCCTGCACCACCA | 57.18 | 18 | 76 |
|  | AGTCCCTCCACGATGCCAA | 57.32 | 19 |  |
| HPRT1 | GCTGAGGATTTGGAGAAGGTGT | 57.67 | 22 | 94 |
|  | GGCCACCCATCTCCTTCAT | 57.32 | 19 |  |
| β-Actin | CCAACCGTGAGAAGATGACC | 57.45 | 20 | 97 |
|  | CCAGAGGCGTACAGGGACAG | 61.55 | 20 |  |
